# Exploring the Educational Value of Popular Culture in Web-Based Medical Education: Pre-Post Study on Teaching Jaundice Using “The Simpsons”

**DOI:** 10.2196/44789

**Published:** 2023-08-17

**Authors:** Nishaanth Dalavaye, Ravanth Baskaran, Srinjay Mukhopadhyay, Movin Peramuna Gamage, Vincent Ng, Hama Sharif, Stephen Rutherford

**Affiliations:** 1 School of Medicine Imperial College London London United Kingdom; 2 School of Medicine Cardiff University Cardiff United Kingdom; 3 OSCEazy Research Collaborative Cardiff United Kingdom; 4 School of Biosciences Cardiff University Cardiff United Kingdom

**Keywords:** educational innovation, jaundice, medical education, popular culture, web-based teaching

## Abstract

**Background:**

The potential of popular culture as a tool for knowledge delivery and enhancing engagement in education is promising but not extensively studied. Furthermore, concerns exist regarding learning fatigue due to increased reliance on videoconferencing platforms following the COVID-19 pandemic. To ensure effective web-based teaching sessions that maintain attention spans and enhance understanding, innovative solutions are necessary.

**Objective:**

This study aims to evaluate the use of specific popular culture case studies to enhance student engagement in a web-based near-peer teaching session.

**Methods:**

We delivered a web-based teaching session to undergraduate medical students in the United Kingdom. The session included clinical vignettes and single-best-answer questions using characters from “The Simpsons” television show as patient analogies for various causes of jaundice. A pre-post survey, employing a 7-point Likert scale, was distributed to gather data from participants.

**Results:**

A total of 53 survey responses were collected. Participants reported significantly improved understanding of jaundice after the session compared to before the session (median 6, IQR 5-6 vs median 4, IQR 3-4.5; *P*<.001). The majority of participants agreed that the inclusion of “The Simpsons” characters enhanced their knowledge and made the teaching session more memorable and engaging (memorability: median 6, IQR 5-7; engagement: median 6, IQR 5-7).

**Conclusions:**

When appropriately integrated, popular culture can effectively engage students and improve self-perceived knowledge retention. “The Simpsons” characters can be used pedagogically and professionally as patient analogies to deliver teaching on the topic of jaundice.

## Introduction

The COVID-19 pandemic has significantly impacted medical students, leading to a transformation in medical education. Teaching through digitalized platforms became a requirement and, in the aftermath of the pandemic, has become a gradual norm among faculty members. While web-based teaching and blended learning approaches have the potential to effectively educate medical students, there are challenges such as reduced attention span, decreased engagement, and “Zoom fatigue” from excessive videoconferencing [1,2]. As web-based teaching continues to persist, innovative approaches are necessary to ensure the learning needs of students continue to be met efficiently. Popular culture refers to cultural products, practices, beliefs, and objects that are highly prevalent within a certain society [3]. Considering popular culture is an intrinsic element of social and political life that individuals are exposed to daily, there may be interest among educators and researchers in its use as an educational tool.

The evidence supporting the use of popular culture in medical education is still emerging but shows promising results. While there is a paucity of research specifically focused on medical education, anecdotal evidence from related fields suggests potential benefits. Educators from various disciplines have reported that popular culture can make content more relatable for students, facilitate understanding of complex concepts, and generate excitement about learning [4-6]. Entertainment-education is a communication strategy that combines entertainment media with educational content. This approach has been successfully applied in public health campaigns to encourage behavior change [7]. By using popular culture narratives and characters, educational messages can be created in an engaging and entertaining format, leading to increased message recall and influence. Certain types of video games, which may incorporate popular culture elements, have also been shown to have positive effects on various health outcomes [8]. In the context of medical education, where students often face challenges in knowledge retention, stressful learning environments, and high-stakes examinations, the potential benefits of popular culture can be particularly valuable [9].

This study focuses on jaundice, which is characterized by yellow discoloration of the skin and sclera [10]. Understanding jaundice is crucial for medical students, as it may indicate underlying liver disease or biliary obstruction requiring urgent investigation [10]. However, comprehending jaundice can be challenging due to its various causes and complex diagnostics [10]. In this study, we explore the educational value of popular culture by using characters from the iconic television show “The Simpsons” to teach jaundice. “The Simpsons” is notorious for its plethora of fictional characters that have a distinctive yellow appearance, potentially resembling the clinical presentation of jaundice. We adopted a near-peer teaching (NPT) approach, which has previously proven successful in a web-based format [2,11].

The purpose of this study was to examine the perceptions of undergraduate medical students regarding the value of using “The Simpsons” characters, and identify potential opportunities for incorporating popular culture into web-based teaching sessions. We hypothesized that attendees would experience high levels of engagement and relatability to the teaching content.

## Methods

### Study Design

This observational study used a pre-post study design to evaluate the impact of incorporating “The Simpsons” characters in teaching jaundice to medical students. It was conducted according to the STROBE (Strengthening the Reporting of Observational Studies in Epidemiology) statement [12].

### Setting

The teaching session, titled “Jaundice for Finals” by OSCEazy, was delivered on the internet through the Zoom platform. It was taught by a fourth-year medical student (ND) to undergraduate medical students in the United Kingdom. The teaching session was delivered in January 2022. The session consisted of a series of clinical vignettes based on the topic of jaundice, including single-best-answer questions to assess knowledge. “The Simpsons” characters were used as patient analogies in the clinical vignettes to provide context for different causes of jaundice. Attendees had the opportunity to answer the single-best-answer questions during the session using the polling function on Zoom. The clinical vignettes and single-best-answer questions used in the session are outlined in Multimedia Appendix 1. The session included focused teaching on each specific cause of jaundice, covering pathophysiology, clinical features, investigations, and management. Previous knowledge of “The Simpsons” television show was not essential to correctly answering the single-best-answer questions. Students were informed at the beginning of the session that the names of the individuals in the clinical vignettes were all “The Simpsons” characters.

### Participants

Since the spring of 2020, we have presented a series of web-based NPT sessions as part of a national student-led teaching initiative called “OSCEazy.” This teaching session was advertised across different social media platforms and was open to health care students worldwide. Participants could access the Zoom meeting link free of charge. The target demographic of the teaching session was students in their clinical years of medical school; hence, the session was advertised as “Jaundice for Finals.” The fact that “The Simpsons” characters were being used in the teaching session was not included in the social media marketing hence attending students could maintain an open perspective on its use. Only completed survey responses from participants studying at an undergraduate medical school in the United Kingdom were included for statistical analysis.

### Outcome Measures

The main outcome assessed in the survey was the participants’ self-perceived improvement in understanding of the topic of jaundice after attending the teaching session. The survey also assessed the participants’ self-perceived improvement in memorability and engagement, as well as whether the addition of “The Simpsons” characters enhanced the overall learning experience compared to if no characters were used.

### Statistical Methods

An optional survey, which consisted of 7-point Likert questions, was distributed at the beginning and end of the teaching session using the chat function on Zoom. The survey was generated using Google Forms and pretested before dissemination (Multimedia Appendix 2). The results of the survey were processed automatically into an Excel (Microsoft Corp) spreadsheet. The Shapiro-Wilk test was used to test the normality of the data distribution. The Wilcoxon matched-pair signed rank test was used to determine statistical significance (*P*<.05). A Spearman rank correlation coefficient was also performed as a nonparametric measure of correlation. Statistical analysis was performed using SPSS (version 28; SPSS Inc). Figures were created using Excel.

### Ethical Considerations

According to advice obtained from the National Health Service Health Research Authority’s web-based decision tool, the study did not require formal ethics committee approval. Students were told on the survey that, upon completion, they consented to the use of their data in future publications. No participants received any type of compensation for their participation. The collected data were anonymized and stored according to the General Data Protection Regulation (GDPR).

## Results

**Figure 1:**
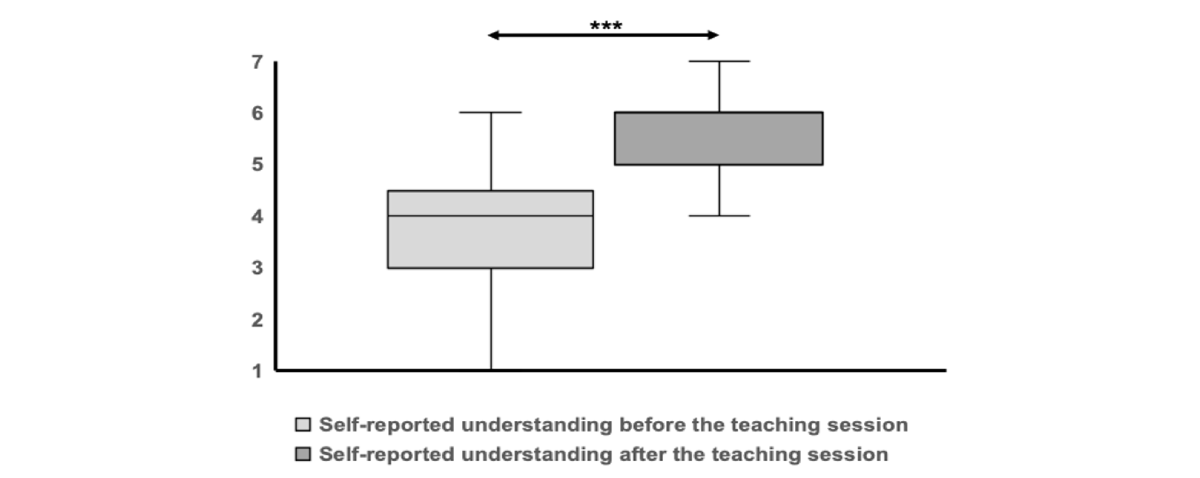
Participants’ self-reported understanding of jaundice before and after the teaching session. The boxes represent the IQR. The horizontal line within the box represents the median value. The whiskers represent the minimum and maximum values. *P<.05; **P<.01; ***P<.001.

A total of 53 attendees completed the optional survey. Overall, 47 (89%) participants were in year 3 of medical school or above. Before the session, 49 (93%) participants had heard of “The Simpsons.” Participants’ self-reported understanding of jaundice after the session was significantly higher than the self-reported understanding of jaundice before the session (median 6, IQR 5-6 vs median 4, IQR 3-4.5; *P*<.001; *R*=0.38; Figure 1). Participants also agreed that the addition of “The Simpsons” characters made the teaching more memorable and engaging (memorability: median 6, IQR 5-7; engagement: median 6, IQR 5.5-7). Participants mostly agreed that the addition of “The Simpsons” characters made the overall learning experience greater than if no characters were included (median 6, IQR 5-7).

## Discussion

### Overview

This study aimed to explore the educational value of using popular culture, specifically characters from the television show “The Simpsons,” in teaching jaundice to medical students. The results of this study provide valuable insights into the potential benefits of incorporating popular culture into web-based medical education. Overall, the findings suggest that using “The Simpsons” characters as patient analogies in the teaching session positively influenced students’ learning experience and understanding of jaundice. Participants reported a significant improvement in their understanding of jaundice and considered the session engaging and memorable.

The advantages of incorporating popular culture may be explained by targeting the affective domain of students’ brain while providing a recognizable mental scenery to explore scientific principles [13]. The use of popular culture aligns with principles of educational psychology, such as schema theory and situated learning [14,15]. By tapping into students’ existing knowledge and experiences with popular culture, educators can help them construct mental frameworks for understanding new information. This connection to familiar contexts can enhance learning and promote deeper comprehension. Visual mnemonics, such as the use of visual cues and imagery, have been shown to improve memory retention and recall [16]. Popular culture references can serve as visual cues that trigger associations and facilitate memory retrieval. By linking medical concepts to recognizable characters or scenarios from popular culture, educators can enhance students’ ability to remember and apply the learned material. The value of visual mnemonics has also been previously demonstrated in the Picmonic Learning System, which improved both memory retention and exam performance [17]. The renowned “The Simpsons” characters were used in a comparable manner to help students especially retain knowledge of the pathophysiology of the different causes of jaundice; the taught information was contextualized based on the notable character personalities and memorable storylines.

The use of popular culture in medical education has several advantages. First, it can help make complex medical concepts more relatable and understandable. By using familiar characters and situations, students can better grasp the nuances of jaundice and its various causes. This approach enhances knowledge retention and promotes active learning. By using popular culture scenarios as case studies, educators can bridge the gap between theoretical knowledge and real-world application. This approach helps students connect medical concepts to practical situations, improving their problem-solving skills and clinical reasoning [18]. Moreover, incorporating popular culture into medical education can generate excitement and increase student engagement. The use of “The Simpsons” characters captured students’ attention and made the learning experience more enjoyable. This engagement is crucial in combating web-based learning challenges, such as reduced attention spans and “Zoom fatigue.” The NPT approach used in this study also proved effective in a web-based format. Having students learn from their peers who have a deeper understanding of the subject matter creates a supportive and relatable learning environment. The use of “The Simpsons” characters further facilitated this peer-to-peer connection and enhanced the overall learning experience.

While this study focused on jaundice, the findings have broader implications for medical education. Popular culture can be used to teach other medical topics and foster a deeper understanding of complex concepts. Incorporating elements of popular culture into web-based teaching sessions has the potential to create a more interactive and engaging learning environment, ultimately improving knowledge acquisition and retention among medical students. A clear concern pertains to the potentially negative connotations of students’ perceptions of the credibility of delivered teaching. This has likely been a major determinant in medical faculty being reluctant to use popular culture considering the exacting standards of professionalism expected [19]. As this teaching session was delivered using an NPT approach and was not regulated by any governing body, it could be argued the session leader had fewer external barriers to incorporating popular culture. Nonetheless, we feel professionalism was maintained due to the primary focus on the clinical knowledge being taught and popular culture being sparingly used as an adjunctive teaching tool.

Popular culture in medical education can have positive effects on promoting empathy and challenging stereotypes. By exposing students to diverse ethnicities, genders, or socioeconomic backgrounds of patients in popular culture, it can encourage a broader understanding of different perspectives [20]. This exposure can help break down stereotypes and unconscious biases that may exist among students, leading to more inclusive patient-centered care. Popular culture also has the potential to offer role models in medicine and challenge traditional stereotypes. Portraying strong and diverse health care professionals in popular culture may inspire aspiring health care providers from underrepresented backgrounds.

It is clear that although the use of popular culture can be pedagogically impactful, its success is highly dependent on the preparation and expertise of the educator. The use of popular culture references also has issues relating to inclusivity, especially regarding learners from diverse cultures and backgrounds. From an educator’s perspective, difficulty will arise in choosing an appropriate popular culture case for the intended audience. In addition to their “jaundice-like” appearance, “The Simpsons” characters were specifically chosen as the current age demographic of UK medical students suggested a large proportion of attendees would have engaged with these characters in their youth. “The Simpsons” are also an internationally recognized brand and should therefore be recognizable outside of the United Kingdom and anglophone context. Using an NPT approach possibly presented an advantage in helping choose a popular culture case, as there is wide consensus that the benefits of NPT stem from the social and cognitive congruence between educators and learners [21]. In addition to this congruence allowing educators to tailor their teaching to an appropriate level, educators are more familiar with popular culture cases that would be appealing to students and can successfully incorporate popular culture.

This preliminary study had some limitations in addition to the small sample size and observational nature, meaning no definitive conclusions can be drawn about the educational value of popular culture. This study was limited in scope to undergraduate medical students in the United Kingdom. Further research is needed to examine the applicability and effectiveness of using popular culture in medical education across different cultural contexts and educational settings. The survey used preset questions with Likert scales, illustrating a closed-ended approach. Although this allowed quantitative analysis of outcomes, it may have driven bias that an open-ended approach with free-text and thematic analysis of responses might have avoided. The analyzed cohort was not controlled, as participants attending were from different universities. More detailed demographic parameters, such as socioeconomic status, were not captured. Although most attendees had previous awareness of “The Simpsons,” the question of whether this previous awareness presented a learning advantage is not fully clear, as their baseline level of exposure and awareness was not quantitatively characterized. Further studies should evaluate baseline knowledge of the relevant popular culture case and robustly assess its long-term impact on knowledge retention and clinical performance.

It is crucial for educators to become proficient in web-based teaching, especially in the current state of academia. Despite the small sample size, these preliminary results suggested that the students responded positively to this novel concept of enhancing web-based teaching sessions and provided a strong initial basis for educators to explore this methodology within their own teaching capacity. However, a teaching session without the amalgamation of popular culture cases was not performed as a comparator. Therefore, further evidence is required before widespread adoption is advocated, as it is currently difficult to ascertain the magnitude of this benefit in comparison to traditional teaching without inclusion of popular culture. The implementation of popular culture should be done thoughtfully, considering factors such as cultural relevance, inclusivity, and professionalism. We implore educators to continually share their unique experiences of how they incorporate different popular culture cases for the universal betterment of web-based education. Tutors should especially explore the value of popular culture in a near-peer setting, where a more informal approach is often desired by students and the social and cognitive congruence may allow more tailored feedback to be garnered.

### Conclusions

This study demonstrates that popular culture, exemplified by “The Simpsons” characters, can be a valuable educational tool in web-based medical education. By leveraging popular culture, educators can enhance student engagement, improve understanding of complex medical concepts, and create a more enjoyable and effective learning experience. The integration of popular culture into medical education has the potential to transform the way medical students learn and retain knowledge.

## Appendix

Multimedia Appendix 1 Single-best answer questions used in teaching session.

Multimedia Appendix 2 Survey used in teaching session.

## Abbreviations

NPT: near-peer teaching

## Data Availability

The data sets generated and analyzed during this study are available from the corresponding author upon reasonable request.
