# Supplementary material for: Exploring the Educational Value of Popular Culture in Web-Based Medical Education: Pre-Post Study on Teaching Jaundice Using “The Simpsons”
Source: JMIR Med Educ. 2023 Aug 17;9:e44789. doi: 10.2196/44789 (PMC10472169; doi:10.2196/44789)
Supplement: Multimedia Appendix 1 [file mededu_v9i1e44789_app1.docx]

**Clinical vignettes**

**Question 1:**

Bart Simpson has demolished his school classroom and has been put in detention. He is forced to write lines on the blackboard as punishment for his actions. Principal Skinner asks him to write what substance is responsible for the brown pigmentation of faeces.

What should he write?

1. Stercobilin
2. Unconjugated bilirubin
3. Urobilinogen
4. Conjugated bilirubin
5. Bile salts

**Question 2:**

Grampa Simpson presents to the GP with a two-month history of painful, numb, blue fingers, and toes. He recently started taking cold showers because of the stress caused by his family. The symptoms are worse after showers but return to normal after he warms up indoors. On examination, he has scleral icterus. His full blood count shows a significantly raised lymphocyte count and there are smudge cells and spherocytes on a blood film.

What is the best investigation to diagnose the likely cause of his jaundice?

1. Flow cytometry
2. Direct antiglobulin test
3. Bone marrow biopsy
4. Osmotic fragility test
5. Cytogenetics

**Question 3:**

Moe has been feeling very down about his lack of a love life and feels his heart is 'broken'. After performing various tests, the doctors recommended him to have a valve replacement and the surgery was performed with no complications. A few weeks later, he begins to develop shortness of breath on exertion. His haemoglobin is 75 g/l and he has scleral icterus. He has a metallic click sound during S2 and there are schistocytes on his blood film.

Which of the following blood test findings is most likely to be seen?

1. ↑ conjugated bilirubin
2. ↑ unconjugated bilirubin
3. Splenomegaly
4. ↑ haptoglobin
5. High mean corpuscular volume

**Question 4:**

Krusty has been asked by his GP to perform a fasting glucose test to check if he has Type 2 Diabetes. He has a history of alpha-thalassemia trait. He has been very stressed all night as he is worried about all his gambling debts. When he comes in, the GP notices he is clinically jaundiced. There are no other notable examination findings.

What is the likely cause of his jaundice

1. Haemolytic crisis
2. Fasting-induced cholestasis
3. Non-alcoholic fatty liver
4. Gilbert’s syndrome
5. Acute chest crisis

**Question 5:**

Apu has had another child born into his family. The baby is 20 hours old and is being reviewed on the ward because of neonatal jaundice . She was born at term following an uncomplicated pregnancy. She has passed meconium. She is alert, well perfused, feeding normally, and afebrile. She is jaundiced over face, trunk, and limbs. Blood tests show an elevated unconjugated bilirubin, normal liver function tests, and no evidence of haemolysis.

What is the most likely cause of jaundice in this newborn?

1. Rhesus incompatibility
2. Sepsis
3. Gilbert’s syndrome
4. Physiological jaundice
5. Crigler-Najjar syndrome

**Question 6:**

Maggie, a 2-month-old infant, is brought to the hospital because of jaundice noted by her family members. She has been otherwise well with normal growth and development. Marge says she has been exclusively breastfeeding her, but she now has very pale stools. Homer is worried that Maggie has something serious which means that he might have to pay more medical expenses. Blood tests show an elevated conjugated bilirubin.

What is the most appropriate next step?

1. Liver biopsy
2. Colonoscopy
3. Reassure and discharge
4. Abdominal ultrasound
5. Sweat chloride test

**Question 7:**

Homer, a 50-year-old alcoholic man, presents to the emergency department because of an episode of hematemesis. He is disoriented to time and place but says this is normal for him. Abdominal examination shows abdominal distension with shifting dullness and caput medusae. There is a bilateral “flapping” tremor, red palms, and bilateral lower limb oedema.

Which of the following is the most likely cause of the patient’s red palms?

1. High levels of ammonia in the blood
2. High levels of bilirubin in the blood
3. Low levels of albumin in the blood
4. High levels of oestrogen in the blood
5. Portal hypertension

**Question 8:**

Sideshow Bob has spent the last thirty years in a foreign prison. After he is released, he comes to the occupational health department for a check-up. Viral hepatitis serology is requested and shown below.

HBs Ag: Positive

Anti-HBc IgM: Negative

Anti-HDV IgM: Positive

Anti-HBc IgG: Positive

Anti-HBs: Negative

Which of the following is the patient's most likely current hepatitis status?

1. Hepatitis B & D co-infection
2. Immunised against hepatitis B virus
3. Chronic Hepatitis B infection
4. Acute Hepatitis B infection
5. Hepatitis B & D superinfection

**Question 9:**

Selma, a 55-year old woman presents with jaundice and mild abdominal pain. She is a heavy-smoker and has smoked 50 cigarettes every day for the past 30 years. She tried to quit smoking once but relapsed after 10 minutes. She has lost over 10kg in the past 2 months. An ultrasound shows a 5cm mass in the head of the pancreas. An ERCP with cytologic sampling shows evidence of malignant cells.

Which of the following tumour markers is used to assess response to treatment?

1. Alpha-fetoprotein
2. Lipase
3. Ca 19-9
4. Ca 125
5. Alkaline phosphatase

**Question 10:**

Marge, a 46-year-old woman, comes to the GP because of pruritus for the past 2 months. She also complains of fatigue but puts this down to having to take care of her kids and Homer. She has a previous history of pain and pallor of her fingers during cold weather. On examination, there is scleral icterus. Blood tests show elevated liver enzymes, particularly GGT and ALP, and elevated conjugated bilirubin. An abdominal ultrasound is normal.

Which of the following antibodies is most likely to be positive?

1. Anti-Scl 70
2. Anti-dsDNA
3. Anti-mitochondrial
4. Anti-smooth muscle
5. P-ANCA
