# Supplementary material for: Exploring the Educational Value of Popular Culture in Web-Based Medical Education: Pre-Post Study on Teaching Jaundice Using “The Simpsons”
Source: JMIR Med Educ. 2023 Aug 17;9:e44789. doi: 10.2196/44789 (PMC10472169; doi:10.2196/44789)
Supplement: Multimedia Appendix 2 [file mededu_v9i1e44789_app2.docx]

Using 'Simpsons' characters to teach jaundice to medical students

Thank you for attending this teaching session. Please fill out this form to help us see what was good about this session. All data will be stored according to GDPR guidelines. By filling in this short form, you are providing consent for use of your data in an anonymised fashion for any future academic publication.

Taking part is completely voluntary. By filling this questionnaire your are giving your consent to participate in this study and for use of your anonymised data for future publication. Any responses you provide are anonymous. Participating in this survey indicates that you are consenting to the handling of data in accordance with Article 6(1)(a) General Data Protection Regulation (GDPR) principles in the UK. Data will be held for a period of 1 year for the purposes of comparing trends and analytics according to the Cardiff University Policy. Access to the data is only granted to the main authors. The data will be used for academic publications and will be stored in a password-protected account.

- What is your current year of medical school?
  - Year 1
  - Year 2
  - Year 3
  - Year 4
  - Year 5
  - Year 6
  - Not in medical school currently
- What was your understanding of jaundice before this session? (Scale = 1-7 where 1=poor and 7=excellent)
- What was your understanding of jaundice after this session? (Scale = 1-7 where 1=poor and 7=excellent)
- Have you heard of 'The Simpsons' TV show before this session?
  - Yes
  - No
- To what extent do you agree with the statement: 'The addition of 'Simpsons' characters improved my knowledge in the topics taught'? (Scale = 1-7 where 1=strongly disagree and 7=strongly agree)
- To what extent do you agree with the statement: 'The addition of 'Simpsons' characters made the information taught more memorable'? (Scale = 1-7 where 1=strongly disagree and 7=strongly agree)
- To what extent do you agree with the statement: 'The addition of 'Simpsons' characters made the overall learning experience greater than if no characters were included? (Scale = 1-7 where 1=strongly disagree and 7=strongly agree)
